# Persistent Severe Acute Respiratory Syndrome Coronavirus 2 Pneumonia in Patients Treated With Anti-CD20 Monoclonal Antibodies

**DOI:** 10.1093/ofid/ofad464

**Published:** 2023-09-15

**Authors:** Nadav Furie, Michal Mandelboim, Neta Zuckerman, Ana Belkin, Lior Seluk, Inbal Shafran, Ronen Mass, Liran Levy, Sumit Chatterji, Erik Baltaxe, Michael Peled, Tiberiu Shulimzon, Abraham Avigdor, Sharon Amit, Amir Onn, Edith M Marom, Galia Rahav, Michael J Segel

**Affiliations:** Institute of Pulmonology, Sheba Medical Center, Tel Hashomer, Israel; Sackler Faculty of Medicine, Tel-Aviv University, Tel Aviv, Israel; Sackler Faculty of Medicine, Tel-Aviv University, Tel Aviv, Israel; Central Virology Laboratory, Public Health Services, Ministry of Health and Sheba Medical Center, Tel-Hashomer, Israel; Sackler Faculty of Medicine, Tel-Aviv University, Tel Aviv, Israel; Central Virology Laboratory, Public Health Services, Ministry of Health and Sheba Medical Center, Tel-Hashomer, Israel; Internal Medicine D, Sheba Medical Center, Tel Hashomer, Israel; Infectious Diseases Unit, Sheba Medical Center, Tel Hashomer, Israel; Institute of Pulmonology, Sheba Medical Center, Tel Hashomer, Israel; Sackler Faculty of Medicine, Tel-Aviv University, Tel Aviv, Israel; Institute of Pulmonology, Sheba Medical Center, Tel Hashomer, Israel; Sackler Faculty of Medicine, Tel-Aviv University, Tel Aviv, Israel; Institute of Pulmonology, Sheba Medical Center, Tel Hashomer, Israel; Institute of Pulmonology, Sheba Medical Center, Tel Hashomer, Israel; Sackler Faculty of Medicine, Tel-Aviv University, Tel Aviv, Israel; Institute of Pulmonology, Sheba Medical Center, Tel Hashomer, Israel; Institute of Pulmonology, Sheba Medical Center, Tel Hashomer, Israel; Sackler Faculty of Medicine, Tel-Aviv University, Tel Aviv, Israel; Institute of Pulmonology, Sheba Medical Center, Tel Hashomer, Israel; Sackler Faculty of Medicine, Tel-Aviv University, Tel Aviv, Israel; Institute of Pulmonology, Sheba Medical Center, Tel Hashomer, Israel; Sackler Faculty of Medicine, Tel-Aviv University, Tel Aviv, Israel; Division of Hematology and Bone-Marrow Transplantation, Sheba Medical Center, Ramat Gan, Israel; Microbiology Laboratory, Sheba Medical Center, Tel Hashomer, Israel; Institute of Pulmonology, Sheba Medical Center, Tel Hashomer, Israel; Sackler Faculty of Medicine, Tel-Aviv University, Tel Aviv, Israel; Sackler Faculty of Medicine, Tel-Aviv University, Tel Aviv, Israel; Department of Diagnostic Radiology, Sheba Medical Center, Tel Hashomer, Israel; Sackler Faculty of Medicine, Tel-Aviv University, Tel Aviv, Israel; Infectious Diseases Unit, Sheba Medical Center, Tel Hashomer, Israel; Institute of Pulmonology, Sheba Medical Center, Tel Hashomer, Israel; Sackler Faculty of Medicine, Tel-Aviv University, Tel Aviv, Israel

**Keywords:** antibody therapy, COVID19, immune globulins, monoclonal antibody, SARS-CoV-2

## Abstract

We report 8 cases of persistent severe acute respiratory syndrome coronavirus 2 (SARS-CoV-2) pneumonia in patients previously treated with anti-CD20 monoclonal antibodies. Polymerase chain reaction of nasopharyngeal swabs for SARS-CoV-2 was negative in most cases; viral cell cultures confirmed that viable SARS-Co-2 virus was present. Four patients were treated with anti-SARS-CoV-2 hyperimmune globulins with rapid resolution of disease.

In most patients with coronavirus disease 2019 (COVID-19), the severe acute respiratory syndrome coronavirus 2 (SARS-CoV-2) viral load decreases over the first week; however, viral ribonucleic acid can be detected for >2 months in immunocompromised hosts [1]. Persistent SARS-CoV-2 infection has been reported in a patient treated with B-cell-depleting anti-CD20 treatment [2] and in a recipient of chimeric antigen receptor T-cell therapy after bone marrow transplantation [3]. We report 8 cases of persistent COVID-19 pneumonia in patients previously treated with anti-CD20 monoclonal antibodies (MAbs).

## METHODS

All adult patients diagnosed at our hospital on May 2021–January 2022 with persistent COVID-19 pneumonia and who had a history of treatment with anti-CD20 MAbs (rituximab or obinutuzumab) were included in the study. Persistent COVID-19 pneumonia was defined as persistence, for >1 month after initial diagnosis of SARS-CoV-2 infection, of all the following:

(1) respiratory and/or systemic symptoms; (2) bilateral pulmonary opacities demonstrated on imaging of the chest; (3) and polymerase chain reaction (PCR) and/or viral cell culture identification of SARS-CoV-2 in an upper or lower respiratory tract sample. The study was approved by the Institutional Ethics Committee. Diagnostic strategy and virology methods are described in detail in the Supplementary material.

### Patient Consent

The requirement for informed consent was waived by the Institutional Review Board due to the retrospective and observational nature of the study.

## RESULTS

Eight cases (5 women, age 34–78 years) were identified. Table 1 summarizes the patients’ demographics and clinical features. Seven patients received anti-CD20 MAb for B-cell malignancies and 1 for myasthenia gravis. All patients had received multiple doses of anti-CD20 MAbs. The time elapsed from the last dose of anti-CD20 until the first PCR-positive nasopharyngeal swab (NPS) ranged from 1 week to 13 months (median, 1.5 months). Clinical details of the acute stage of COVID-19 are provided in the Supplementary material.

**Table 1.** Patient Demographics, COVID-19 Timeline, Clinical Features, Treatment, and Follow up

Patient No.	1	2	3	4	5	6	7	8
Age	60	62	46	67	78	68	34	41
Sex	M	F	M	F	M	F	F	F
Underlying immunosuppression	CLL	PCNSL	FL	FL	MZL	FL	MG	FL
Treatment with anti-CD20 MAb	O	Ri	O	O	Ri	Ri	Ri	Ri
SARS-CoV-2 Vaccination (doses)^a^	0	0	0	2	0	0	1	0
Symptoms								
Dyspnea	+	+	+	+	+	+	+	+
Fever	+	−	+	+	+	+	−	−
Cough	−	−	−	+	+	−	−	+
Weight loss	−	−	−	+	+	−	−	+
Date of COVID-19 diagnosis (1st positive NPS-PCR)	1/2021	1/2021	11/2020	7/2021	2/2021	8/2021	7/2021	08/2021
Time from last anti-CD20 treatment to 1st positive SARS CoV-2 NPS	1 wk	1 m	1 m	13 m	3 m	4 m	1.5 m	1 wk
Time from 1st positive NPS to diagnosis of persistent COVID-19	5 m	5 m	7 m	4 m	7 m	2 m	6 m	7 m
Time from last anti-CD20 treatment to diagnosis of persistent COVID-19	6.2 m	5 m	7.5 m	17 m	10 m	6 m	7.5 m	7.2 m
Hyperimmune globulin treatment	Yes	No	No	Yes	Yes	No	Yes	No
GCS	Yes	No	No	Yes	No	Yes	Yes	No
Convalescent plasma	No	No	No	No	Yes	No	No	No
Antivirals	R	No	No	No	R	No	No	S, P
Other	−	−	−	−	−	S	−	−
Follow-up	6 m	8 m	4 m	8 m	2 m (died)	6 m	6 m	NA
Clinical improvement	Yes	Yes	Yes	Yes	No	Yes	Yes	NA
Radiological improvement	Yes	Yes	Yes	Yes	No	No	Yes	NA
CRP (mg/L)	3.7	3.6	NA	14	1.8	17	4.8	1.2
NPS-PCR	+	−	NA	−	−	+	+	NA

Abbreviations: CLL, chronic lymphocytic leukemia; COVID-19, coronavirus disease 2019; FL, follicular lymphoma; GCS, systemic glucocorticosteroids; m, months; MG, myasthenia gravis; MZL, marginal zone lymphoma; NA, not available; Neg, negative; NPS, nasopharyngeal swab; O, obinutuzumab; P, Paxolovid; PCNSL, primary central nervous system lymphoma; R, Remdesivir; Ri, rituximab; S, sarilumab; wk, weeks.

NOTE: Treatment refers to treatment given after a diagnosis of persistent COVID was made.

a All vaccines were BNT162b2.

**Figure 1.**
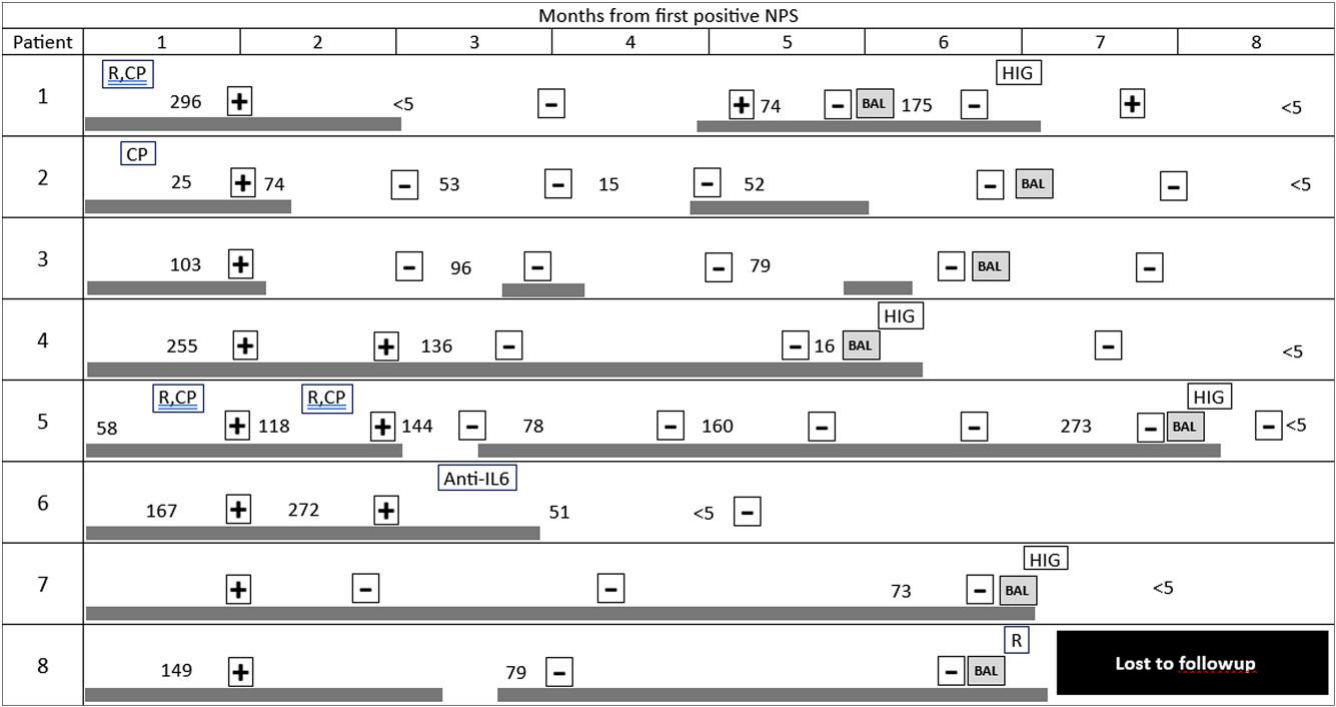
Timeline (in months) of events for individual patients, starting at the time of the first nasopharyngeal swab (NPS) positive for SARS-CoV-2 (by PCR) (first positive NPS not otherwise indicated). Horizontal gray bars denote periods in which the patient was symptomatic. Numbers denote serum C-reactive protein (CRP) values in milligrams per liter (upper limit of normal, 5 mg/L). +, positive repeat NPS; −, negative repeat NPS; anti-IL6, humanized anti-interleukin 6 receptor monoclonal antibody; BAL, positive BAL; CP, treatment with convalescent plasma; HIG, treatment with Kamada anti-SARS-CoV-2 hyperimmune globulin; R, treatment with remdesivir.

Persistent SARS-CoV-2 pneumonia was diagnosed 2–7 months after the first PCR-positive NPS. The time-course of individual patients is depicted in Figure 1. During these months patients reported symptoms including cough, dyspnea, fever, night sweats, and weight loss (Table 1), with a generally indolent clinical course. All had been hospitalized for investigation, including laboratory testing and imaging. All had been treated with courses of antibiotics. Three patients were treated with glucocorticosteroids (0.5–1 mg/kg per day prednisone); they experienced a relapse of symptoms when steroids were tapered.

At the time of diagnosis of persistent COVID-19, all patients were anemic; 6 were lymphopenic; and all had elevated C-reactive protein (CRP) (Supplementary Table 1). Bronchoalveolar lavage (BAL) fluid cultures (7 patients) for bacteria, mycobacteria, and fungi and PCR for *Pneumocystis jirovecii*, cytomegalovirus, and respiratory viruses were negative. The BAL cell differential count (5 patients) revealed prominent lymphocytosis (28%–72%). Transbronchial biopsies performed in 4 patients demonstrated nonspecific interstitial pneumonia with predominantly lymphocytic infiltrate. Neither BAL cytology nor biopsies had cytopathic changes.

Chest radiographs showed unilateral or bilateral opacities that migrated on follow up (Supplementary Figure 1*A* and *B*). Chest computed tomography (CT) scans of all patients showed waxing and waning bilateral ground-glass opacities (Supplementary Figure 1*C*).

Repeated NPS PCR for SARS-CoV-2 were negative or borderline (cycle threshold [Ct] ≥35) in all but 1 patient (no. 6). In contrast, PCR of BAL fluid was positive (Ct < 30) in all 7 patients in whom BAL was performed. In addition, 3 patients had positive SARS-CoV-2 viral cell culture from BAL fluid, confirming viable virus was present in the lower respiratory tract. Viral sequencing showed that patients were infected with different SARS-CoV-2 variants—Alpha (B.1.1.7), Delta (B.1.617.2, AY.121, AY.4.13), and other SARS-CoV-2 lineages that circulated in Israel before the emergence of the Alpha variant (B.1.1.50, B.1.1.294). The same variant was detected in all nasopharyngeal samples collected from each patient. In some samples, the variant detected was no longer circulating in Israel at the time of sampling, indicating that infection had occurred much earlier. Viral genome sequence comparison from repeated positive samples showed the emergence and accumulation of mutations over time, within structural genes such as the spike, nucleocapsid, and membrane and in ORF genes (Supplementary Table 2).

One patient was lost to follow up; the rest were followed for 2–8 months (Table 1). Three patients (nos. 2, 3, and 6) experienced spontaneous clinical improvement. Three months after diagnosis of persistent COVID-19, 1 patient (no. 2) had a CT scan that showed resolution of lung opacities. Repeat BAL fluid was negative for SARS-CoV-2 by both PCR and culture, which had been positive previously. The CT scans of patients nos. 3 and 6 two months after the diagnosis of persistent COVID-19 still showed bilateral opacities. One patient (no. 8) received nirmatrelvir/ritonavir for persistent infection, with symptomatic improvement and normalization of CRP. Four patients who continued to be symptomatic (nos. 1, 4, 5, and 7) were treated with Kamada-Anti-SARS-CoV-2 hyperimmune globulins (details in Supplementary material) and experienced resolution of symptoms (fever and dyspnea) within 2 weeks. Three patients who had been dependent on oral prednisone for weeks were successfully tapered off prednisone after treatment with hyperimmune globulins. Two of these patients normalized restrictive defects on pulmonary function tests. The CRP decreased in all 4 patients after treatment (Table 1). Opacities on chest radiographs resolved. Three patients had CT scans 1–3 months after treatment, showing complete resolution of lung opacities. Posttreatment NPS PCR was negative in 2 of the 4 treated patients; the other 2 had borderline-positive swabs up to 2 months posttreatment. To date, several months after treatment, all 4 treated patients remain asymptomatic.

One patient (no. 5) died during follow up. He had improved significantly after treatment with hyperimmune globulins. Two months later, bone morrow aspiration revealed transformation to leukemia. He died 1 week later. Cause of death determined by the treating physicians was leukemia, not related to COVID-19 infection or to hyperimmune globulins.

## DISCUSSION

We present 8 cases of persistent SARS-CoV-2 pneumonia in patients who were immunosuppressed by prior treatment with anti-CD20 antibodies, mostly for B-cell malignancy. Our case definition required a combination of symptoms consistent with the diagnosis, chest imaging demonstrating pulmonary opacities, and identification of SARS-CoV-2 in a respiratory sample.

In most of our patients, NPS-PCR was negative, and the diagnosis required BAL fluid testing, consistent with previous reports [4] that NPS-PCR has poor sensitivity for the diagnosis of persistent SARS-CoV-2 pneumonia. Our findings suggest that SARS-CoV-2 may persist in the lower respiratory tract of immunosuppressed hosts long after the upper respiratory tract has been cleared of virus.

To the best of our knowledge, ours is the first report of persistent SARS-CoV-2 confirmed by viral cell culture. Viral cell culture confirms the presence of viable virus, and it is very specific but less sensitive than PCR, as known from other viruses [5]. In patients clinically recovered from COVID-19, NPS-PCR can remain positive, whereas viral culture is negative [6], suggesting either that viral culture for SARS-CoV-2 is less sensitive than PCR or that PCR may detect nonviable virus. The BAL testing may be more sensitive than NPS and may have a role in patients with a high clinical suspicion for COVID-19 who test negative by NPS-PCR [7].

Viral mutations accumulated over time in the patients in our report; these mutations are potentially a source for variant generation, which could spread in the population at large. In previous reports, time to diagnosis of persistent SARS-CoV-2 pneumonia was ≤3 months [4, 8]. In our patients, persistent SARS-CoV-2 pneumonia was diagnosed as long as 7 months after the first positive NPS-PCR, and as long as 17 months after the last treatment with anti-CD20 MAb, consistent with reports that immune deficiency after anti-CD20 treatment can last for more than 1 year [9]. Our report highlights the critical role of humoral immunity in clearing the lower respiratory tract of SARS-CoV-2. This concept is supported by a report of prolonged time to SARS-CoV-2 clearance from the respiratory tract in patients with X-linked agammaglobulinemia [10].

All of our patients were evaluated and treated with courses of antibiotics and/or glucocorticosteroids for several months before the diagnosis of persistent COVID-19 pneumonia was made. A high index of suspicion is the key to timely diagnosis. When persistent COVID-19 infection is suspected, and NPS-PCR is negative, BAL should be performed and tested for SARS-CoV-2.

Four of our patients were treated with anti-SARS-CoV2 hyperimmune globulins with apparently positive effect. It is notable that 3 other cases in our series experienced spontaneous clinical improvement, although 2 of them had persistent opacities on follow-up chest imaging. In a recent, randomized, controlled trial in patients hospitalized with symptomatic COVID-19, hyperimmune globulins failed to show efficacy when added to standard care [11]. Only 5% of participants in that study were immunosuppressed. Further studies of the role of hyperimmune globulins in the treatment of COVID-19 seem warranted, particularly in persistent COVID-19 pneumonia in immunocompromised hosts. The main limitations of our report are the retrospective observational nature and the small number of cases.

## CONCLUSIONS

In conclusion, patients previously treated with anti-CD20 MAbs may develop SARS-CoV-2 pneumonia persisting for many months. Clinicians caring for patients with a history of exposure to anti-CD20 MAbs should have a high index of suspicion for persistent viral pneumonia. Sampling the lower respiratory tract by BAL is crucial to the diagnosis. We suggest the following diagnostic criteria for persistent COVID-19 pneumonia: (1) a probable diagnosis of persistent SARS-CoV-2 pneumonia should be based on the combination of respiratory symptoms, pulmonary opacities, and BAL positive for SARS-CoV-2 by PCR; (2) a definite diagnosis requires a positive viral cell culture from a lower respiratory sample. Finally, our experience suggests treatment with hyperimmune globulins should be further investigated in persistent SARS-CoV-2.

## Supplementary Material

ofad464_Supplementary_Data

## Supplementary Data

Supplementary materials are available at *Open Forum Infectious Diseases* online. Consisting of data provided by the authors to benefit the reader, the posted materials are not copyedited and are the sole responsibility of the authors, so questions or comments should be addressed to the corresponding author.
