# Supplementary material for: Persistent Severe Acute Respiratory Syndrome Coronavirus 2 Pneumonia in Patients Treated With Anti-CD20 Monoclonal Antibodies
Source: Open Forum Infect Dis. 2023 Sep 15;10(10):ofad464. doi: 10.1093/ofid/ofad464 (PMC10551847; doi:10.1093/ofid/ofad464)
Supplement: ofad464_Supplementary_Data [file ofad464_supplementary_data.zip › ORIF Supp R2.docx]

**SUPPLEMENTAL INFORMATION**

**METHODS**

***PATIENTS AND DIAGNOSTIC TESTING***

Patients with suspected persistent COVID19 underwent repeat nasopharyngeal RT-PCR testing, SARS-CoV-2 serologic testing, viral genome sequencing, viral culture and bronchoalveolar lavage (BAL) with or without biopsy. BAL was performed to rule out other etiologies for diffuse lung opacities in immunocompromised patients, including [*Pneumocystis jirovecii*](https://www.google.co.il/search?safe=active&hl=iw&sxsrf=ALiCzsaVnVK2RvVhfVEvVA--zYD5EsXBUQ:1658264525077&q=pneumocystis+jirovecii&spell=1&sa=X&ved=2ahUKEwigyNff7IX5AhUS16QKHZ7vABkQkeECKAB6BAgBEDw)*,* CMV, fungi, mycobacteria, and other respiratory viruses and bacteria.

**VIROLOGY METHODS**

Nasopharyngeal swabs (NPS) were collected, and RT-PCR testing was performed by Allplex 2019-nCoV assay (Seegene Inc., S. Korea), with results expressed as the cycle threshold (Ct) for the gene encoding the nucleocapsid protein (N gene), according to the manufacturer's instructions.

To identify variants of concern, we performed multiplex real-time one-step RT-PCR assays to detect mutations in the spike (S) protein (E484K, N501Y, and HV69/70). To verify the results, whole-genome sequencing was performed with the use of the preparation kit (Illumina, San Diego, CA, USA), as described previously [1].

For viral culture Vero E6 cells were cultured at 37°C until reaching 70% confluence. Inoculation was performed using samples that tested positive for SARS-CoV-2 in the PCR assay. The culture was incubated for 1 hour at 33⁰C with 300 µl of the filtered BAL samples, followed by addition of 5 ml 2% FCS MEM-EAGLE medium. The infected cells were cultured for 7 days and were inspected visually for the onset of cytopathic effect (CPE). Upon CPE onset, supernatants were collected, RNA was extracted, and RT-PCR testing was performed as described above [2]. Cultures were considered positive when the Ct value of the culture supernatant was lower (indicating more virus) than that of the original BAL fluid.

Serum was tested for IgG antibodies against SARS-CoV-2 RBD using the commercial automatic chemiluminescent microparticle immunoassay SARS-CoV-2 IgG II Quant (Abbott Laboratories, Abbott Park, IL, USA) according to the manufacturer’s instructions.

***SARS-CoV-2 whole genome sequencing and bioinformatics analysis***

The COVID-seq kit was used for library preparation according to the manufacturer’s instructions (Illumina). Library validation and mean fragment size was determined by TapeStation 4200 via DNA HS D1000 kit (Agilent, Santa Clara, CA, USA). Libraries were pooled, denatured, and diluted to 10pM and sequenced on NovaSeq (Illumina).

Fasq files underwent quality control using FastQC (www.bioinformatics.babraham.ac.uk/projects/fastqc/) and MultiQC [3]. The Burrows-Wheeler aligner (BWA mem) [4] was used to map reads to the SARS-CoV-2 reference genome (NC_045512.2), followed by filtering unmapped reads, sorting, and indexing of the resulting BAM files using the SAMtools suite [5] and forming a consensus FASTA using iVAR (https://andersen-lab.github.io/ivar/html/index.html). SARS-CoV-2 variant and mutation detection was done using a custom pipeline (https://github.com/NetaZuckerman/covid19) utilizing Nextclade (https://clades.nextstrain.org/) and Pangolin (https://cov-lineages.org/resources/pangolin.html).

***PLASMA-DERIVED HYPERIMMUNE GLOBULINS***

Anti-SARS-CoV-2 (Kamada, Kibbutz Bet Kama, Israel) is a preparation of immune gamma globulins manufactured from convalescent plasma of COVID19 recovered patients, using proprietary purification methods. Patients received a single dose of 4 gr. intravenously [6].

**RESULTS – ADDITIONAL INFORMATION**

In the **acute stage of COVID19** five patients were hospitalized, four of them due to severe disease necessitating supplemental oxygen. Hospitalized patients received low molecular weight heparin and dexamethasone, three were treated with remdesivir and convalescent plasma, and one was treated with anti-IL6 MAB. Two patients who were not hospitalized had received a course of dexamethasone. Only 2 patients had been vaccinated with SARS COV2 vaccine (BNT162b2).

**Table S1.** Active immunomodulatory treatment while diagnosed with persistent COVID 19 infection

| **Patient number** | **Treatment** |
| --- | --- |
| **1** | Venetoclax, Obinutuzumab |
| **2** | Rituximab, Cytarabine |
| **3** | Obinutuzumab |
| **4** | - |
| **5** | Bendamustine+Rituximab |
| **6** | Rituximab |
| **7** | Rituximab |
| **8** | Rituximab, Venetoclax |

**Table S2.** Laboratory results at time of diagnosis of persistent COVID19

| Patient # | 1 | 2 | 3 | 4 | 5 | 6 | 7 | 8 |
| --- | --- | --- | --- | --- | --- | --- | --- | --- |
| Diagnostic test for persistent COVID19 | BAL PCR & Culture | BAL PCR & Culture | BAL PCR & Culture | BAL PCR | BAL PCR | NPS | BAL PCR | BAL PCR |
| BAL PCR Ct / concurrent NPS Ct ^1^ | 28 / Neg | 29 / Neg | 28 / Neg | 26 / 38 | 30/ Neg | NA | 24/ 35 | 22/ Neg |
| Anti-SARS-CoV-2 Ab | 0 | 0 | 0 | 0 | 3 AU/ml | 184 BAU/ml | NA | NA |
| Serum IgM (mg/dl) | <19 | NA | 21 | NA | NA | NA | 73 | NA |
| Peripheral Blood CD20 | NA | NA | 0 | 0 | 0 | NA | NA | NA |
| Hemoglobin  (g/dL) | 10.9 | 11.1 | 9.0 | 10.7 | 8.2 | 8.0 | 8.8 | 11.2 |
| WBC  (K/microL) | 7.6 | 9.8 | 3.3 | 4.6 | 0.7 | 11.0 | 8.0 | 2.9 |
| Neutrophils  (K/microL) | 5.9 | 5.3 | 2.3 | 2.8 | 0.56 | 10.8 | 6.24 | 1.32 |
| Lymphocytes  (K/microL) | 0.70 | 3.4 | 0.54 | 0.90 | 0.13 | 0.22 | 0.64 | 1.20 |
| ALK P (U/L) | 85 | 98 | 86 | 105 | 77 | 126 | NA | NA |
| ALT (U/L) | 55 | 10 | 22 | 15 | 7 | 41 | NA | NA |
| AST (U/L) | 47 | 19 | 39 | 32 | 13 | 55 | NA | NA |
| LDH (U/L) | 257 | NA | 397 | 381 | 306 | 575 | NA | NA |
| Creatinine (mg/dL) | 0.66 | 0.74 | 0.55 | 0.53 | 0.45 | 0.41 | NA | NA |
| CRP (mg/L) | 132 | 50 | 79 | 101 | 233 | 130 | 59 | 78 |

Results obtained within 1 week of diagnosis of persistent COVID19.

Hgb – Hemoglobin; WBC – Total white blood cell count; ALK P – Alkaline phosphatase, ALT – Alanine transaminase; AST – Aspartate transaminase; LDH – Lactate dehydrogenase; CRP – C-reactive protein; NA – not available; NPS – nasopharyngeal swab.

**Table S**3 Virus Genotyping

| **Patient** | **Sample site** | | **Sample date** | | **Variant** | **Mutations (nucleotides)** | **Mutations (amino acid replacements)** |
| --- | --- | --- | --- | --- | --- | --- | --- |
| #1 | | NPS | | 17/08/2021 | B.1.1.50 | A1699T, A4233C, C23895T, T24055C, A25866C, A2903G, T5260A, C9438T,  C12781T, C12789T, C14220T, C19610T, G22708T, G26063T, C26645T, G29260T | ORF1ab:D1323A, ORF1ab:I880V, ORF1ab:T3058I, ORF1ab:T4175I,  ORF1ab:T6449I, S:T778I, ORF3a:G224V |
| #2 | | NPS | | 16/02/2021 | B.1.1.294 | C5549T, C25366T, C745T, T5260A | ORF1ab:L1762F |
|  | | NPS | | 21/02/2021 | B.1.1.294 | C5549T, C25366T, G29477T, C17135T, C22522T | ORF1ab:L1762F, N:D402Y, ORF1ab:P5624L |
|  | | NPS | | 01/02/2021 | B.1.1.294 | C5549T, C25366T, G29477T, C17135T, C22522T, A2903G | ORF1ab:L1762F, N:D402Y, ORF1ab:P5624L, ORF1ab:I880V |
|  | | BAL | | 02/09/2021 | B.1.1.294 | C5549T, C25366T, G29477T, T1783C, A4905G, C9924T, G12355A,  A20658G, C21627T, C22311A, C22550T, C26333T, A26802C, A28249G | ORF1ab:L1762F, N:D402Y, ORF1ab:D1547G, ORF1ab:A3220V,  S:T22I, S:T250N, S:P330S, E:T30I, M:S94R, ORF8:D119G |
| #3 | | BAL | | 06/06/2021 | B.1.1.50 | A1638G, A3139G, G3302A, C3903T, C4763T, G4866T, A5459C, T7114A, A12978G,  T13474C, T23042C, C23604T, C24026T, C26333T, C27335T, C2306T, C4230T, C4795T,  G5180A, C5730T, C8905T, T18417C, C18647T, A20262G, C21789T, C26895T, C29523T | ORF1ab:K458R, ORF1ab:E1013K, ORF1ab:P1213L, ORF1ab:H1500Y,  ORF1ab:S1534I, ORF1ab:M1732L, ORF1ab:N4238S, ORF1ab:C4404R,  ORF1ab:L681F, ORF1ab:T1322I, ORF1ab:D1639N, ORF1ab:T1822I,  ORF1ab:P6128L, S:S494P, S:P681L, S:L822F, S:T76I, E:T30I, M:H125Y,  ORF6:T45I, N:T417I |
| #4 | | NPS | | 31/10/2021 | NA | low coverage |  |
|  | | BAL | | 04/11/2021 | AY.4.13 (Delta) | C3365T, A14801C, T22200C, C19951T, G28048T, A29782G | ORF1ab:L1034F, ORF1ab:D4846A, ORF1ab:P6563S, S:V213A, ORF8:R52I |
| #5 | | NPS | | 12/02/2021 | B.1.1.50 | C2455T, G3663A, A4800G, A18801G, C18912A, C22088T, T5260A, G26526T | ORF1a: S1133N, ORF1a:K152R, ORF1b:D1815E, S:L176F, M:A2S |
|  | | NPS | | 19/02/2021 | B.1.1.50 | C2455T, G3663A, A4800G, A18801G, C18912A, C22088T, G8179A, C16466T | ORF1a: S1133N, ORF1a:K152R, ORF1b:D1815E, S:L176F, ORF1b:P1000L |
|  | | NPS | | 03/03/2021 | B.1.1.50 | C2455T, G3663A, A4800G, A18801G, C18912A, C22088T, T5182G,  C15017T, C26204T, A28271T | ORF1a: S1133N, ORF1a:K152R, ORF1b:D1815E, S:L176F |
|  | | NPS | | 24/03/2021 | B.1.1.50 | C2455T, G3663A, A4800G, A18801G, C18912A, C22088T | ORF1a: S1133N, ORF1a:K152R, ORF1b:D1815E, S:L176F |
|  | | NPS | | 27/03/2021 | B.1.1.50 | C2455T, G3663A, A4800G, A18801G, C18912A, C22088T, G8179A, C16466T,  C5184T, T5260A, G22319A, A2905G | ORF1a: S1133N, ORF1a:K152R, ORF1b:D1815E, S:L176F,  ORF1b:P1000L, ORF1a:P1640L, S:D253N, ORF1a:I880M |
|  | | NPS | | 28/03/2021 | B.1.1.50 | C2455T, G3663A, A4800G, A18801G, C18912A, C22088T, T5182G, C15017T,  C26204T, A28271T, T5260A, C29085T | ORF1a: S1133N, ORF1a:K152R, ORF1b:D1815E, S:L176F,  S:L141-143del, N:T271I |
|  | | NPS | | 29/03/2021 | B.1.1.50 | C2455T, G3663A, A4800G, A18801G, C18912A, C22088T, G8179A, C16466T,  C5184T, T5260A, G22319A | ORF1a: S1133N, ORF1a:K152R, ORF1b:D1815E, S:L176F, ORF1b:P1000L,  ORF1a:P1640L, S:D253N |
|  | | NPS | | 05/04/2021 | B.1.1.50 | C2455T, G3663A, A4800G, A18801G, C18912A, C22088T, T5260A, C29085T,  A2903G, A18233G, T26837G, T26861G | ORF1a: S1133N, ORF1a:K152R, ORF1b:D1815E, S:L176F, S:L141-143del,  N:T271I, M:N113K, ORF1a:I880V, ORF1b:Q1589R |
|  | | NPS | | 13/04/2021 | B.1.1.50 | C2455T, G3663A, A4800G, A18801G, C18912A, C22088T, T5182G, C15017T,  C26204T, A28271T, T5260A, C2880T, G24776C | ORF1a: S1133N, ORF1a:K152R, ORF1b:D1815E, S:L176F, S:L141-143del,  ORF1a:A872V, S:E1072Q |
| #6 | | NPS | | 18/09/2021 | AY.121 (Delta) | A2903G, T8672C | ORF1ab:I880V, ORF1ab:S2803P |
|  | | NPS | | 20/10/2021 | AY.121 (Delta) | A2903G, T8672C | ORF1ab:I880V, ORF1ab:S2803P |
|  | |  | |  |  |  |  |
|  | |  | |  |  |  |  |

NPS – nasopharyngeal swab

**Figure S1. Typical chest radiograph and computed tomography of persistent COVID19 infection**

Chest radiographs of a typical patient (#1) one month apart (a,b), with consolidation (arrows) migrating between the studies. The chest CT (c) of patient #3 demonstrates bilateral scattered heterogeneous opacities, mainly of ground glass density, but also the reverse halo sign, which sometimes correlates to organizing pneumonia in histopathology (curved arrows).

**References**

1. Zuckerman NS, Pando R, Bucris E, et al. Comprehensive analyses of SARS-CoV-2 transmission in a public health virology laboratory. Viruses 2020;12:854-854.
2. Jefferson T, Spencer EA, Brassey J, Heneghan C. Viral cultures for COVID-19 infectious potential assessment — a systematic review. Clin Infect Dis 2020 December 3
3. Ewels P, Magnusson M, Lundin S, Käller M. MultiQC: summarize analysis results for multiple tools and samples in a single report. Bioinformatics. 2016 Oct 1;32(19):3047-8.
4. Li H, Durbin R. Fast and accurate short read alignment with Burrows-Wheeler transform. Bioinformatics. 2009;25(14):1754-1760.
5. Handsaker B, Wysoker A, et al. The Sequence Alignment/Map format and SAMtools. Bioinformatics. 2009;25(16):2078-2079.
6. Hyperimmune anti SARS-CoV-2 immunoglobulin concentrate in hospitalized patients with COVID-19 pneumonia: results of an open label phase I/IIA clinical trial. Yuval Ishaya, Yasmin Maorb, et al. Submitted to publication
