# Supplementary figures and images for: Persistent Severe Acute Respiratory Syndrome Coronavirus 2 Pneumonia in Patients Treated With Anti-CD20 Monoclonal Antibodies
Source: Open Forum Infect Dis. 2023 Sep 15;10(10):ofad464. doi: 10.1093/ofid/ofad464 (PMC10551847; doi:10.1093/ofid/ofad464)

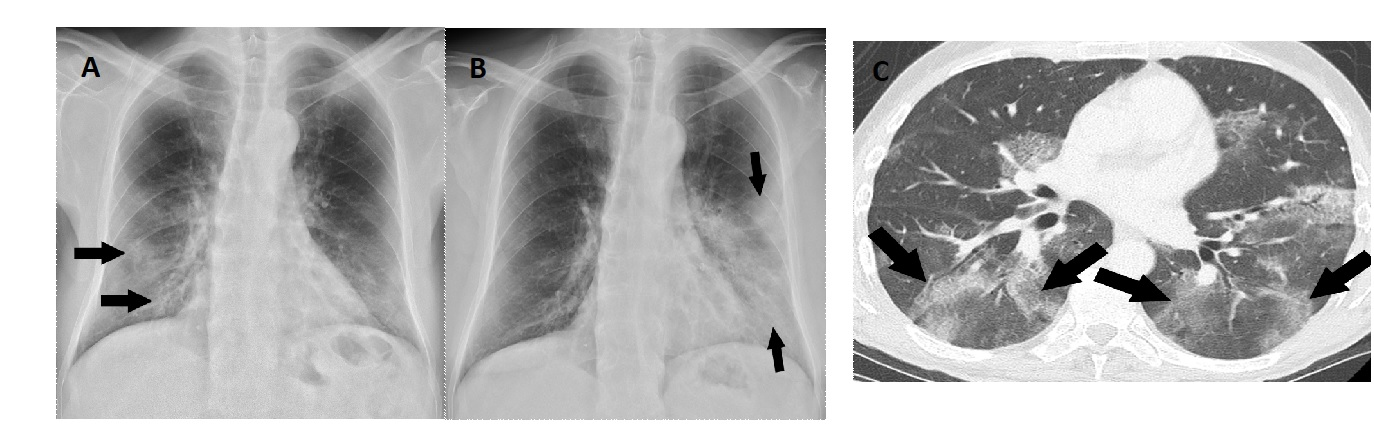

Supplement: ofad464_Supplementary_Data [file ofad464_supplementary_data.zip › Figure S1 600DPI.tif]
